# Nurses in the Male Wards of Lunatic Asylums

**Published:** 1904-07-09

**Authors:** 


### EDITOR'S LETTER-BOX.

[Our Correspondents are reminded that prolixity is a great bar to publication, and that brevity of style and conciseness of statement greatly facilitate early insertion.]

### NURSES IN THE MALE WARDS OF LUNATIC ASYLUMS.

AN "Old Medical Superintendent" writes:—In your article in this week's HOSPITAL on the above, you write that "for 20 years or more it has been customary to employ nurses in the men's infirmary wards of some asylums." If you had written 50 years instead of 20, you would have been nearer the mark. That great pioneer of improvement in the management of asylums, Lockhart Robertson, to the writer's knowledge, had nurses in the mens infirmary at the County Asylum for Sussex at Hayward's Heath as far back as 1860. It was tried for several years by Robertson and his successor, but eventually dropped as the work was found to be too revolting and laborious for any but most exceptionally constituted females.

